# Dopamine, Cognitive Impairments and Second-Generation Antipsychotics: From Mechanistic Advances to More Personalized Treatments

**DOI:** 10.3390/ph13110365

**Published:** 2020-11-05

**Authors:** Sebastiano Alfio Torrisi, Samuele Laudani, Gabriella Contarini, Angelina De Luca, Federica Geraci, Francesca Managò, Francesco Papaleo, Salvatore Salomone, Filippo Drago, Gian Marco Leggio

**Affiliations:** 1 Department of Biomedical and Biotechnological Sciences, University of Catania, 95123 Catania, Italy; sebastiano.torrisi@unict.it (S.A.T.); s.laudani91@hotmail.it (S.L.); gabriella.contarini@unict.it (G.C.); angelinadeluca17@gmail.com (A.D.L.); geraci.federica@icloud.com (F.G.); salomone@unict.it (S.S.); f.drago@unict.it (F.D.); 2 Genetics of Cognition laboratory, Neuroscience area, Istituto Italiano di Tecnologia, 16163 Genova, Italy; Francesca.Manago@iit.it (F.M.); Francesco.Papaleo@iit.it (F.P.)

**Keywords:** cognition, schizophrenia, dopamine receptors, second-generation antipsychotics

## Abstract

The pharmacological treatment of cognitive impairments associated with schizophrenia is still a major unmet clinical need. Indeed, treatments with available antipsychotics generate highly variable cognitive responses among patients with schizophrenia. This has led to the general assumption that antipsychotics are ineffective on cognitive impairment, although personalized medicine and drug repurposing approaches might scale down this clinical issue. In this scenario, evidence suggests that cognitive improvement exerted by old and new atypical antipsychotics depends on dopaminergic mechanisms. Moreover, the newer antipsychotics brexpiprazole and cariprazine, which might have superior clinical efficacy on cognitive deficits over older antipsychotics, mainly target dopamine receptors. It is thus reasonable to assume that despite more than 50 years of elusive efforts to develop novel non-dopaminergic antipsychotics, dopamine receptors remain the most attractive and promising pharmacological targets in this field. In the present review, we discuss preclinical and clinical findings showing dopaminergic mechanisms as key players in the cognitive improvement induced by both atypical antipsychotics and potential antipsychotics. We also emphasize the concept that these mechanistic advances, which help to understand the heterogeneity of cognitive responses to antipsychotics, may properly guide treatment decisions and address the unmet medical need for the management of cognitive impairment associated with schizophrenia.

## 1. Introduction

Schizophrenia is a chronic multifactorial neuropsychiatric disorder with an incidence of 0.7–1% [1]. Patients with schizophrenia experience severe symptoms that are historically allocated in three main symptom clusters: (i) positive symptoms (e.g., delusions, hallucinations); (ii) negative symptoms (e.g., lack of motivation and social withdrawal); (iii) cognitive impairments (impairment across several cognitive domains including memory, attention, and executive functioning) [2].

At a pharmacological level, antipsychotics represent first-line pharmacotherapy for the treatment of schizophrenia [3,4]. In line with the clinical heterogeneity of this disorder, the pharmacological responses to antipsychotics are highly variable [5]. In particular, whereas antipsychotics substantially reduce or eliminate positive symptoms, their effects on negative symptoms and cognitive impairment are highly variable and far from optimal [4,6]. Regarding cognitive impairments associated with schizophrenia (CIAS), no pharmacological treatments have been approved so far by any regulatory agencies worldwide [7,8]. This has produced considerable interest mostly because CIAS have been reported as the main cause of functional disability and poor quality of life [9]. Classically, antipsychotics fall into two categories: Typical antipsychotic drugs or first-generation antipsychotic (FGAs) drugs, and atypical antipsychotic drugs or second-generation-antipsychotic (SGAs) drugs. There is still a debate if SGAs are superior to FGAs in treating CIAS. Meta-analyses have reported SGAs as being modestly more effective than FGAs in ameliorating CIAS [10,11]. Nevertheless, FGAs may have opposite effects on CIAS. FGAs may indeed worsen CIAS [12]; They may improve CIAS similarly to SGAs at low doses [13]; Sometimes they may also result in being more effective compared to SGAs in ameliorating CIAS [14]. Overall, both FGAs and SGAs, when administered at appropriate doses, may produce an improvement in CIAS, but neither category results in being clinically superior to the other. At a mechanistic level, SGAs generally differ from FGAs for their lower affinity for dopamine D2 receptors and high affinity for serotonin 5-HT2A receptors. However, multiple lines of evidence suggest that the cognitive improvement exerted by old and new SGAs still depends on dopaminergic mechanisms, and it is unclear whether the efficacy of SGAs is linked to serotoninergic mechanisms [15]. In this respect, not only the dopamine D2 receptor (D2R) but also D1, D3, and D4 receptors (D1R, D3R, D4R) have been investigated for their possible contribution in the SGAs-induced cognitive improvement. Among these previously overlooked dopamine receptors, the D3R appears to be highly involved in the cognitive improvement produced by some SGAs and currently represents one the most attractive target for future drug development or repositioning in the context of CIAS pharmacotherapy.

## 2. The Dopamine Hypothesis of Schizophrenia

Dopamine (DA) influences multiple physiological functions, including reward, cognition and emotional processes through two classes of DA receptors—the D1-like receptors (D1R and D5R) and the D2-like receptors (D2R, D3R, and D4R)—which are G-protein-coupled receptors coupled to Gs and Gi protein, respectively [16,17,18,19]. Dopaminergic neurons, which are mainly located in the midbrain, create four major dopaminergic pathways [20]. Dopaminergic neurons of the ventral tegmental area (VTA) give rise to the mesocortical pathway by innervating the prefrontal cortex (PFC). They also give rise to the mesolimbic pathway, sending dopaminergic projections to the ventral striatum. The nigrostriatal pathway consists of dopaminergic projections from the substantia nigra to the dorsal striatum. Lastly, dopaminergic neurons located in the arcuate nucleus of the hypothalamus, which send projections to the median eminence, create the tuberoinfundibular pathway.

The DA hypothesis of schizophrenia, which postulates a dysregulation of dopaminergic pathways in the etiology of the disorder, has been reconceptualised over the last five decades. The first version of DA hypothesis of schizophrenia postulated an overall hyperdopaminergia and then an excess transmission at DA receptors [21,22]. In 1991, Davis and colleagues [23] proposed an updated version (second version) that postulated a frontal hypodopaminergia and a striatal hyperdopaminergia. They specifically hypothesized that the frontal hypodopaminergia caused negative symptoms, whereas the striatal hyperdopaminergia was responsible for the onset of the positive symptoms. In the more recent version (third version), Howes and Kapur [24] hypothesized that interactions between multiple environmental and genetic risk factors lead to a final common pathway of increased presynaptic striatal dopaminergic function. In contrast to the classic mesolimbic dogma of schizophrenia, recent in vivo neuroimaging data suggest that this presynaptic dopaminergic dysfunction is more prominent in the dorsal striatum rather than in the ventral striatum [15,25,26]. Interestingly, this increased dorsal striatal dopaminergic signalling play, a prominent role not only in the development of positive symptoms, but also in the development of the CIAS, mainly by disturbing dopamine-dependent cortical processes [27].

## 3. Dopamine, Cognition and CIAS

DA modulation of bidirectionally interconnected cortico-striatal circuitries is fundamental for the expression of cognitive functions. In accordance with multiple lines of evidence, this modulation appears rather complex. For instance, the relationship between DA and cognition, mainly in the PFC, follows an inverted U-shaped curve, where either high or low DA levels impair performance in cognitive tasks [28,29]. This is further complicated by findings showing variable effects of dopaminergic drugs on the cognition of human subjects, differing for their baseline levels of cognitive performance [30,31]. According to preclinical studies, the effects of dopaminergic drugs may depend on baseline levels of DA in the PFC [32,33], which hosts a large number of DA receptors [34]. Although there is consensus that the stimulation of D1Rs leads to pro-cognitive effects, this is not always the case. Indeed, the administration of a D1R agonist in rodents may improve or impair cognitive performance during difficult and easy tasks, respectively [35,36]. Moreover, an inverted-U shaped response to D1 receptor stimulation has been reported in monkeys, where low doses of the D1R agonist SKF81297 improved spatial working memory and a high dose of the same agonist impaired spatial working memory [37]. With regard to D2R, the administration of either antipsychotics with high affinity for D2R or more selective D2R antagonists have been basically associated with cognitive impairment [12,38,39]. Clinical and preclinical studies have reported that this detrimental effect is related to a disruption of the D2R-mediated signalling in the PFC. In fact, the administration of sulpride impaired the working memory of human volunteers tested in PFC-dependent tasks [40,41]. These results were substantiated by a rodent study, showing an impairment of novel object recognition memory after a selective blockade of D2R in PFC [42]. In this context, much attention has been paid to another member of the D2-like family, the D3R, which appears to have a prominent role in the modulation of PFC-related cognitive functions [43,44]. Indeed, if in the past it was believed that D3R was located only in subcortical regions of the brain. Nowadays, new technological advancements (bacterial artificial chromosome transgenic GFP reporter mice, [45]) have allowed us to discover a cortical localization of D3R and thus a role of this receptor in the homeostasis of PFC. In particular, Clarkson and colleagues demonstrated that the D3Rs are localized in a novel subclass of pyramidal neurons in the layer V of the medial PFC (mPFC), which project their axons toward different cortical and subcortical areas, and are both electrophysiologically and anatomically distinct from adjacent neurons expressing D1Rs or D2Rs [46]. In line with this evidence, D3R modulates PFC-dependent cognitive function, as demonstrated by the fact that either pharmacological or genetic manipulation, able to affect prefrontal D3R, alter cognition [42,47,48,49]. Opposite to D2R, it is well-established that antagonism/partial agonism on D3R produces pro-cognitive effects across species [50].

Although PFC is the brain region most studied in this field, increasing evidence suggests that DA modulates cognition by acting directly within other brain areas, such as the striatum and the hippocampus [51,52,53,54]. Thus, it is not surprising that dysfunctions of dopaminergic signalling are associated with CIAS. Generally, CIAS are thought to originate from a hypofunctionality of the mesocortical dopaminergic pathway [55]. This concept has been widely supported by several lines of research involving either patients or animals. For instance, it has been observed that reduced levels of DA metabolites in the cerebrospinal fluid of schizophrenic patients, correlated with working memory deficits [56]. In line with these findings, a more recent preclinical study reports a significant reduction in DA and DOPAC in the PFC of mice that exhibited schizophrenia like-behaviors, including memory impairment [57]. Noteworthy, only in 2015, after the accumulation of multiple findings indicating a cortical hypodopaminergia in schizophrenia, Slifstein and colleagues provided, for the first time, in vivo evidence for a diminished amphetamine-induced DA release in the dorsolateral PFC of schizophrenic patients, which correlated with working memory-induced activation of the same area [58]. This prefrontal hypodopaminergia leads to an insufficient D1R stimulation, which in turn triggers CIAS [59]. In this regard, a higher dorsolateral PFC D1R availability in drug naïve schizophrenic patients has been observed, which negatively correlated with working memory abilities [60,61]. These findings may be explained as possible compensatory mechanisms in response to blunted D1R-mediated signaling in PFC. It has also been suggested that CIAS may originate from an imbalance between D1R/D2R activation in the PFC [62]. With respect to the D2R, a genetic-driven disruption of D2R-mediated signaling in PFC has been associated with CIAS [63]. Moreover, we recently found that an interaction between D3R and dysbindin, which is a protein that has been specifically linked to CIAS, generates a D2R/D3R imbalance in the PFC [43].

Besides the cortical brain areas, the striatum plays a key role in the modulation of cognitive functions and thus in the pathophysiological mechanisms underlying CIAS [27]. Related to that, the striatal hyperdopaminergic state characterizing schizophrenic patients, which has been historically linked to positive symptoms, is also responsible for the appearance of CIAS [27]. This may be strictly linked to the functional and anatomical connection between the striatum and the cortex. It has indeed reported that an increased postsynaptic D2R-mediated dopaminergic signaling in the striatum may induce a cortical hypodopaminergia, by interfering with the firing pattern of VTA dopaminergic neurons [27,58,64,65]. This concept is supported by several preclinical findings. In particular, the selective increase in D2R availability in the striatum of genetically modified mice, which showed long-lasting cognitive deficits during prefrontal-dependent cognitive tasks relevant to schizophrenia, generated a reduced DA turnover in the PFC [66,67]. As mentioned above, the striatal hyperdopaminergic state in schizophrenia is more restricted to the dorsal part (associative striatum) than to the ventral part of the striatum [15]. With regard to CIAS, this hyperdopaminergic state within the associative striatum has been linked to disrupted decision-making processes observed in schizophrenia [68]. Thus, it is still reasonable to state that therapeutic interventions aimed at reversing these dopaminergic dysfunctions may be fundamental for treating CIAS.

## 4. Potential Antipsychotics or SGAs Targeting Specific Dopamine Receptors May Become the Cornerstone of CIAS Pharmacotherapy

### 4.1. Potential Antipsychotics

In an attempt to overcome the clinical issue related to the problematic pharmacological management of CIAS, several efforts have been made to develop effective drugs targeting DA receptors. Among them, the D1R represents a promising pharmacological target in the context of CIAS [69]. In particular, full D1R agonists may be effective against CIAS, according to what has already been discussed above. Several clinical studies have in fact reported the full D1R agonist dihydrexidine (DAR-0100A) effective in improving CIAS (especially working memory deficits) both in individuals with schizophrenia and in individuals with schizotypal personality disorder [70,71]. However, DAR-0100A, which has a limited pharmacokinetic profile, also failed to improve CIAS in a randomized controlled trial involving patients with schizophrenia, likely because of its lack of D1R occupancy at low doses [72]. This has prompted researchers to develop better D1R agonists able to achieve sufficient D1R occupancy. Related to that, Meltzer and colleagues demonstrated that the allosteric DA D1R potentiator DETQ, was able to counteract the object recognition memory deficits in mice tested in the phencyclidine (PCP) model for the study of schizophrenia [73]. More recently, it has been characterized a novel non-catecholamine DA receptor D1 agonist, PF-6142, which belongs to a new series of D1R-selective non-catechol agonists endowed with optimal pharmacokinetic properties. Intriguingly, PF-6142 improved cognitive deficits in different rodent models for the study of schizophrenia, based on NMDA receptor hypofunction [74].

As discussed before, multiple lines of evidence have underlined the great potential of drugs targeting the D3R for the treatment of CIAS. In this regard, Sun and colleagues proposed a potent D3R antagonist, Y-QA31, as a potential antipsychotic by proving its preclinical efficacy in well-validated models for the study of schizophrenia. Y-QA31, besides its ability to ameliorate MK-801-induced hyperlocomotion, and methamphetamine-induced prepulse inhibition disruption, was shown to be effective on the MK-801-induced impairment of novel object recognition memory [75]. Another potential antipsychotic, F17464, which interestingly, has reached the clinical phases, is characterized by a potent D3R antagonist activity [76]. In rodents, F17464 was more effective in counteracting scopolamine-induced cognitive deficits than other SGAs [44]. More importantly, in a randomized, double-blind, placebo-controlled study, Bitter and colleagues demonstrated the pharmacological efficacy of F17646 in patients with schizophrenia. At a dose of 40 mg, F17464 showed therapeutic efficacy in ameliorating positive and negative symptoms and, especially, CIAS without the occurrence of weight gain or extrapyramidal side effects [77]. The success of this clinical trial might be ascribed to the relationship between the dose and the D3R occupancy. It was indeed reported that, 6–9 h after the administration of 30 mg of F17464, D3R occupancy in the brain is very high (94% on average), and sufficient to induce pro-cognitive effects [78]. Interestingly, a clinical study reported that buspirone, which is an azapirone anxiolytic drug having 5-HT1A partial agonist activity, as well as D3R/D4R antagonist activity [79], co-administered with SGAs, better ameliorated CIAS than SGAs alone in patients with schizophrenia. In this respect, we previously showed that buspirone counteracted MK-801-induced schizophrenia-like phenotypes, including the deficit of temporal order recognition memory, selectively through its D3R antagonism [80]. However, the beneficial effect of buspirone on CIAS needs to be further investigated because it may be more complex through a possible involvement of other neurotransmitter systems [81].

The role of D4R in cognition and in the development of CIAS is unclear and needs to be further investigated. However, there is evidence that D4R stimulation may be a valuable strategy to improve CIAS [82,83]. RP5063 is a potential SGA under development, having peculiar pharmacological proprieties, including partial agonism at D2R, D3R and also D4R. Notably, Rajagopal and colleagues showed that the beneficial effect of acute administration of RP5063 on sub-chronic PCP-induced impairment in novel object recognition memory can be blocked by a pre-treatment with a selective D4R antagonist [84].

### 4.2. Second-Generation Antipsychotic Drugs (SGAs)

As mentioned before, there is still one question to be answered: Are commercially available SGAs effective against CIAS? Here, we review evidence suggesting that some SGAs, through dopaminergic mechanisms, are able to ameliorate CIAS.

#### 4.2.1. Clozapine

Clozapine, the first SGA developed, is basically one of the most effective, although its use is limited because of the possible occurrence of severe adverse effects. The precise clozapine mechanism of action and mainly the mechanisms whereby clozapine exerts beneficial effects, particularly on CIAS [11,12,85,86], are still unclear despite more than 50 years of research [87]. Nevertheless, it is well-known that clozapine is endowed with nanomolar affinity for serotonin receptors (5-HT2A, 5-HT2C, 5-HT_6_, 5-HT_7_) and uniquely for D4R [88]. Evidence supports the idea that the pro-cognitive effects induced by clozapine rely on dopaminergic mechanisms. For instance, the clinical benefits of clozapine on emotional memory deficits observed in schizophrenic patients have been associated to the capacity of clozapine to stabilize DA levels in the amygdala in a dopaminergic state-dependent manner [89]. In line with this, the pro-cognitive effect of clozapine has been further linked to the evidence that this drug restored DA turnover in the dorsolateral PFC, prelimbic cortex, and cingulate cortex of monkeys chronically treated with PCP [90]. In addition, the pro-cognitive effect of clozapine in mice that exhibited PCP-induced cognitive impairment relevant for schizophrenia, depended on the activation of D1R in the PFC [91].

As mentioned above, there is considerable variability in the clinical responses to antipsychotics. This phenomenon may be explained, taking into account genetic variation, namely differences in DNA among patients. Related to that, it is noteworthy to mention a clinical research paper in which Woodward and colleagues discovered an association between a single nucleotide polymorphism (SNP), the val108/158met, within the gene encoding for catechol-O-methyltransferase (COMT), which is an enzyme involved in the control of PFC DA neurotransmission, and the cognitive improvement induced by clozapine. In particular, 6 months of treatment with clozapine induced a cognitive improvement exclusively in met homozygous and val/met heterozygous patients with schizophrenia [92].

#### 4.2.2. Risperidone

Risperidone is an SGA that has high affinity for the 5-HT2A and D2R as well as moderate to low affinity for, D1R, D3R, and D4R receptors [93]. Evidence from translational studies indicates that risperidone improves CIAS, especially in schizophrenic patients with genetic-induced alterations of the cortical dopaminergic signaling. Scheggia and colleagues reported, indeed, a pharmacogenetic interaction between genetic variants associated with reduced dysbindin expression and the cognitive improvement exerted by antipsychotics including risperidone [6]. They discovered that the viral-mediated silencing of D2Rs in the mPFC of dysbindin heterozygous mice abolished the beneficial effect of risperidone on cognitive dysfunctions relevant for schizophrenia. Furthermore, they discovered that this pharmacogenetic interaction triggers an enhancement of presynaptic cortical D2R-mediated signaling through an increased D2S/D2L ratio, which are the functional isoforms of D2R. Along with these findings, we have recently reported that an epistatic interaction, dysbindin/D3R, drives different cognitive improvement after treatment with risperidone. In particular, we demonstrated that chronic treatment with risperidone produced a greater improvement of executive and working memory functions specifically in both schizophrenic patients and genetically modified mice bearing concomitant reduction in D3R and Dys functionality [43].

#### 4.2.3. Aripiprazole

Aripiprazole differs from earlier SGAs in having partial agonist activity at D2R/D3R, 5HT1A and 5HT2C [94]. In this respect, findings from preclinical models utilized for studying CIAS reported that the aripiprazole’s potential for the treatment of CIAS may depend on dopaminergic mechanisms and its partial agonist activity. Fejgin and colleagues indeed suggested that the superior preclinical efficacy of aripiprazole compared to clozapine and olanzapine in counteracting the PCP-induced the impairment of prepulse inhibition [95], which might model the attentional deficits present in treatment-resistant patients with schizophrenia [96], may indeed rely on its partial agonism-induced DA stabilizing effects. Despite the low affinity of aripiprazole for D1R [97], the pro-cognitive effect of this SGA on PCP-induced cognitive impairment in mice, was found to depend on the activation of D1R [98]. At a clinical level, the relationship between D2R/D3R receptor occupancy by aripiprazole and cognition/CIAS has been interestingly investigated. Whereas higher striatal D2R/D3R receptor occupancy by aripiprazole was associated with decreased working memory in healthy volunteers [99], the same higher striatal D2R/D3R receptor occupancy by aripiprazole was instead reported to be positively correlated with cognitive improvement in patients with schizophrenia [100].

#### 4.2.4. Asenapine

Asenapine is also a multitarget SGA with peculiar pharmacological properties. In particular, asenapine has high affinity for serotonin receptors (5HT1A, 5HT1B, 5HT2A, 5HT2C, 5HT6 and 5HT7) and among DA receptors, it has higher affinity for D3R than D2R [101]. In line with the aforementioned data linking D1R activation by SGAs (clozapine and aripiprazole) and the induction of pro-cognitive effects, also in the case of asenapine, its ameliorative effect on PCP-induced memory impairment can be blocked through the administration of a selective a D1R antagonist [102]. This effect might be indeed due to the ability of asenapine to increase dopaminergic neurotransmission and facilitate NMDA-mediated neurotransmission in the mPFC selectively through D1R activation [103]. However, further studies need to be carried out in order to better understand the mechanisms by which asenapine exerts its pro-cognitive effect, also in relation to its preferential binding to D3R, which it is currently a well-established and interesting pharmacological target for CIAS treatment [16,50].

#### 4.2.5. Blonanserin

Blonanserin differs from the earlier SGAs for its equal antagonist activity at D2R and 5HT2A as well as for its potent antagonist activity at D3R [104,105]. Hida and colleagues provided evidence for an involvement of both D1R and D3R in the ameliorating effect of blonanserin on the PCP-induced cognitive impairment of mice. Indeed, this beneficial effect of blonanserin could be blocked by a pretreatment with either a D3R agonist or a D1R antagonist [106]. These findings were further supported by a different study reporting that the blonanserin-induced cortical-striatal acetylcholine, DA, noradrenaline and striatal DA efflux rely selectively on its antagonism on D3, as well as the concomitant ameliorating effect of blonanserin on PCP-induced cognitive impairment [107]. Interestingly, this effect appears to be reproducible across species because it was also reported that blonanserin rescued the executive function deficits induced in marmosets through the administration of a D3R agonist [108].

#### 4.2.6. Cariprazine

Cariprazine is a recently FDA-approved D3R/D2R partial agonist, which preferentially binds to D3R. Among SGAs that behave like partial agonists, cariprazine has the highest affinity for D3R, followed by aripiprazone and bexpiprazole [109,110]. This feature may be responsible for the pro-cognitive effect of cariprazine observed in preclinical studies. It has been indeed observed that cariprazine counteracted PCP-induced impairments of working memory, attention set-shifting, and recognition memory in wild-type mice, but not in D3R knock-out mice [111]. These findings were further strengthened by other preclinical studies showing an ameliorating effect of cariprazine on cognitive dysfunctions relevant for schizophrenia, which were modelled by using different animal models for the study of CIAS [112,113]. At a mechanistic level, this pro-cognitive effect of cariprazine may be linked to its D3R partial agonist activity, which may normalize DA-induced cortical-striatal abnormalities characterizing schizophrenia.

#### 4.2.7. Brexpiprazole

Brexpiprazole is a novel SGA endowed with partial agonist activity at D2R/D3R and 5-HT1A together with antagonist activity at 5-HT2A. There is evidence that the preclinical beneficial effect of brexpiprazole on cognitive impairment relevant for schizophrenia [114], may depend on dopaminergic mechanisms. In fact, brexpiprazole, through the stimulation of D1R, is able to potentiate NMDAR-induced currents and electrically evoked EPSPs in the mPFC, which is a mechanism linked to pro-cognitive effects [115].

## 5. Concluding Remarks and Future Directions

The pharmacological treatment of CIAS is challenging because of the highly variable cognitive responses observed in patients with schizophrenia chronically treated with antipsychotics. According to evidence discussed in this review, these heterogeneous pharmacological responses might be ascribable to genetic variability, which affects dopaminergic signaling, might interfere with individual capacity to recover from CIAS depending on antipsychotic treatment. In this context, several findings discussed in this review demonstrate that selectively targeting the dopaminergic system might be a good strategy for the treatment of CIAS. In this context, we want also to underline that cognitive-enhancing drugs targeting other neurotransmitter systems involved in the pathophysiology of schizophrenia, such as the glutamatergic system, may be useful. These drugs can be used in combination with SGAs, if possible, at low doses, to obtain a better clinical efficacy on CIAS and to lower the occurrence of side effects associated with D2R blockade. Moreover, focusing on the genetics of individual patients and identify novel mechanisms affecting cognitive responses—mainly dopaminergic based—may allow for a better patient stratification and may help to guide the choice of the more appropriate drug. In an age when the low clinical success rate for de novo drug discovery has led several pharmaceutical industries to downscale or close their clinical neuroscience research programs, improving the use of commercially available SGAs or the repositioning of drugs for which a dopaminergic-based pro-cognitive effect has been demonstrated, might be useful to improve the pharmacological management of CIAS in genetically selected patients with schizophrenia.
